# Converting Spent Cu/Fe Layered Double Hydroxide into Cr(VI) Reductant and Porous Carbon Material

**DOI:** 10.1038/s41598-017-07775-8

**Published:** 2017-08-04

**Authors:** Minwang Laipan, Haoyang Fu, Runliang Zhu, Luyi Sun, Jianxi Zhu, Hongping He

**Affiliations:** 1 CAS Key Laboratory of Mineralogy and Metallogeny/Guangdong Provincial Key Laboratory of Mineral Physics and Materials, Guangzhou Institute of Geochemistry, Chinese Academy of Sciences (CAS), Guangzhou, 510640 China; 2 University of Chinese Academy of Sciences, Beijing, 100049 China; 3 Department of Chemical & Biomolecular Engineering, Polymer Program, Institute of Materials Science, University of Connecticut, Storrs, CT 06269 USA

## Abstract

Recycling solid waste as functional materials is important for both environmental remediation and resource recycling. This study attempts to recycle spent Cu/Fe layered double hydroxide (Cu/Fe-LDH) which is generated from the adsorption of dyes by converting to Cr(VI) reductant and porous carbon material. Results showed that the obtained reductant was mainly composed of Fe^0^ and Cu^0^, and exhibited good reductive activity toward Cr(VI). The species of Fe^0^, Fe^2+^, Cu^0^, and Cu^+^ all favored the reduction of Cr(VI) according to X-ray photoelectron spectroscopy analysis. During Cr(VI) removal, solution pH could increase to neutral which caused the metal ions to precipitate near completion. On the other hand, the spent Cu/Fe-LDH could be employed to produce porous carbon materials, and the generated waste metals solution herein could be reused for LDH synthesis. Specific surface areas of the obtained carbon materials varied from 141.3–744.2 m^2^/g with changes in adsorbed amount of dyes on the LDH. This study illustrates that all the components of wastes can be useful resources, offering a simple recycling approach for similar organic-inorganic solid wastes. This work also enlightens us that designing a proper initial product is crucial to make waste recycling simpler.

## Introduction

Layered double hydroxides (LDHs), one type of anionic clays, have been considered as promising adsorbents for organic contaminants due to their high adsorption performance^1–3^, and low cost in synthesizing with tunable metal compositions. These merits make LDHs promising candidates for large-scale applications in organic contaminants treatment, such as textile dye removal. However, one serious challenge is that an abundance of organic-inorganic solid wastes (i.e., the spent/used LDHs) will be produced. Without proper disposal, these solid wastes may cause secondary environmental pollution and resource waste. Efforts to remedy this issue need to be made.

Conventionally, the most widely used methods for the disposal of organic-inorganic solid wastes are landfilling, cropland application, and incineration. Even though these methods have their own advantages, they present great drawbacks as well. For instance, they can respectively cause poisonous and unmanageable landfill leachate^4^, disgusting odor, soil contamination by heavy metals^5^, and malodorous flue gases^6^. Due to the drawbacks of the conventional methods, a more effective and safe alternative need be developed.

The most promoted alternative to deal with organic-inorganic waste is waste recycling, which is deemed as one of the most innovative solutions in waste disposal due to the great environmental and economic benefits^7^. According to Wei *et al*. and Xue *et al*., the spent Mg/Al-LDH adsorbents can be recycled as nanofiller/colorant filler for polymer materials^8, 9^. These studies indicate that the spent LDHs can be converted to functional materials. Our preliminary results also showed that the spent LDH with regular metal compositions (e.g., Mg and Al) could be used as a superior pore-forming template for preparing curled sheet-like porous carbon material^10^, and spent LDH with transition metal (e.g., Fe and Ni) compositions could be utilized to synthesize magnetic catalysts^11^. In the preliminary study, one interesting phenomenon was observed, being that some of the high valence metal ions (Fe^3+^ and Ni^2+^) were reduced to lower valence by the adsorbed organic contaminants under a pyrolysis procedure; but due to the limited amount of adsorbed organic contaminants, the reduction was only partially achieved. The preliminary results enlighten us that the spent LDHs can possibly be used as a precursor to simultaneously prepare reductant and porous carbon materials. Thanks to the tunability of the metal compositions of LDHs, one can design various types of LDH with high adsorptive performance and reducible metal compositions. These types of LDHs may benefit both environmental remediation and waste resource recycling due to the ease of converting such spent LDHs to functional materials.

**Figure 1:**
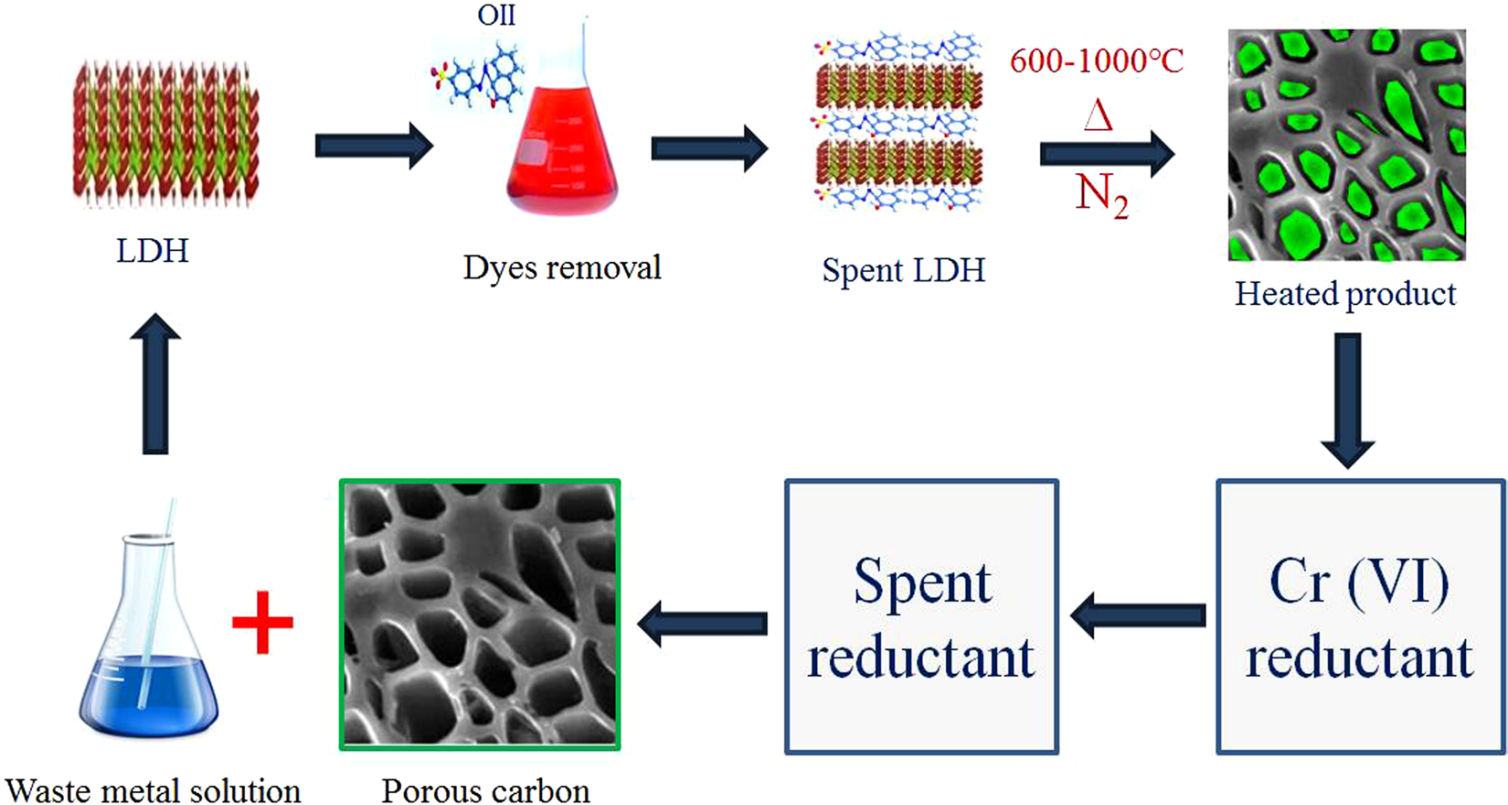
Cyclic utilization of LDH and LDH-type organic-inorganic solid wastes.

This study tried to synthesize one kind of LDH with high adsorptive performance toward organic contaminants and metal compositions reducible by the adsorbed organic matters. Also this study intended to test the possibility of preparing reductant and porous carbon materials from the spent LDH, the main procedures of this work are illustrated in Fig. 1. Cu^2+^ and Fe^3+^ were chosen to construct Cu/Fe-LDH, and an anionic dye Orange II (hereinafter referred to as OII) was used for an organic contaminant. The highly toxic heavy metal hexavalent chromium (Cr(VI)) was employed as a probe to investigate the reducing ability of the generated reductant. The adsorption property of Cu/Fe-LDH toward OII was first examined, and then the spent Cu/Fe-LDH was collected to prepare the complex of reductant and porous carbon material. X-ray diffraction (XRD), thermogravimetric analysis (TG), and N_2_ adsorption-desorption methods were utilized to investigate the structural properties of the obtained products. Meanwhile, Cr(VI) reduction experiments were carried out to evaluate the reducing activity of the generated complex, and X-ray photoelectron spectroscopy (XPS) was employed to explore the Cr(VI) removal mechanism. This work not only provides a simple approach for recycling spent LDHs as functional materials, but also shows us that via designing a proper initial product, one can easily recycle the generated waste.

## Results and Discussion

### Textural characterization results of Cu/Fe-LDH and its OII adsorption performance

**Figure 2:**
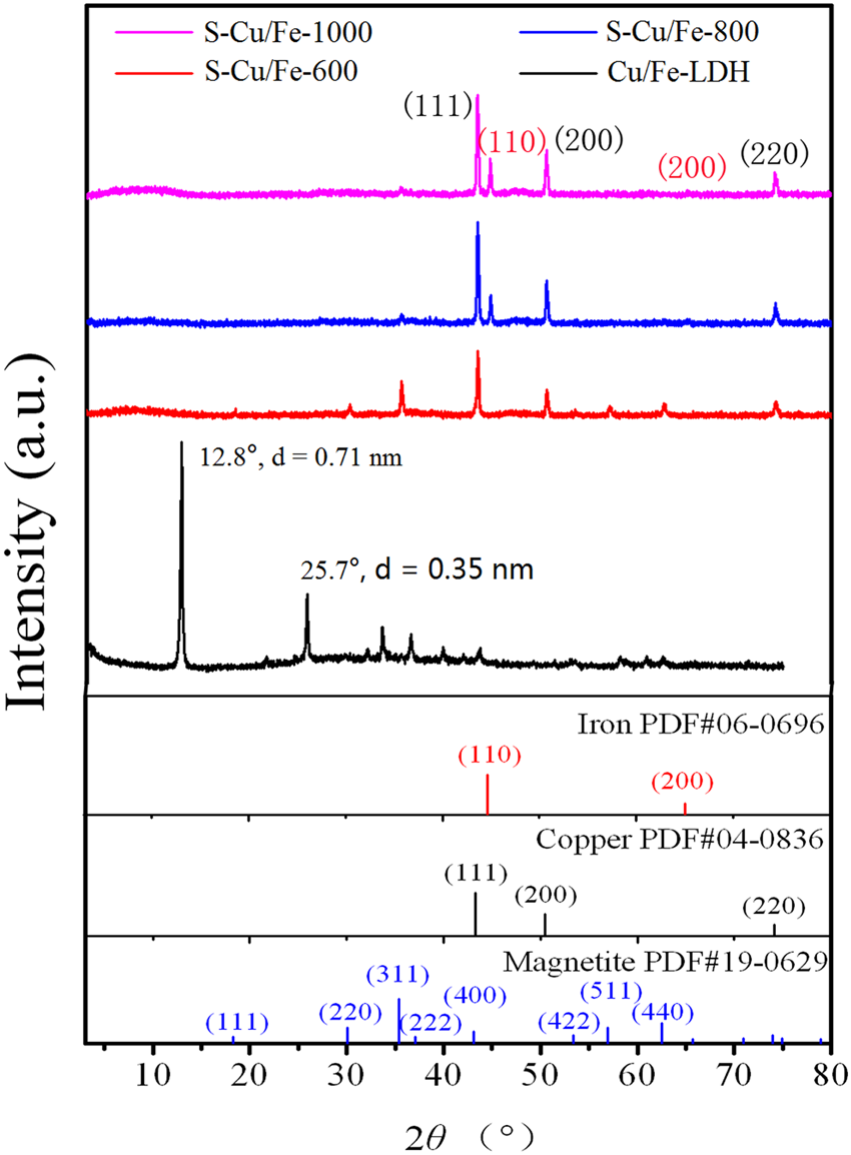
XRD patterns of Cu/Fe-LDH and the heated products of spent Cu/Fe-LDH.

The XRD pattern showed that Cu/Fe-LDH was well crystallized with a typical layered structure as the d-value of the first strong peak at 12.8° (0.71 nm) was twice of that of the second strong peak at 25.7° (0.35 nm) (Fig. 2). SEM characterization result further demonstrated the forming of layered structures (Fig. s1; Supplementary Materials). The adsorption isotherms for OII adsorbed by Cu/Fe-LDH can be considered as a Langmuir type (Fig. s2), indicating a strong affinity between OII and Cu/Fe-LDH at the adsorption sites. Cu/Fe-LDH presented a highly saturated surface containing adsorbed OII (>800 mg/g), which is likely due to the easy exchangeability of the counterion (i.e., NO_3_^−^) in the interlayers of LDH^12^. The replacement of NO_3_^−^ with OII was verified by the XRD pattern as the basal spacing increased (Fig. s3). The high crystallinity and adsorption ability might make Cu/Fe-LDH attractive in dye removal, and then, spent LDH rich in organic contaminants would be generated during the dye removal process. To obtain the raw experimental material (i.e., the spent Cu/Fe-LDH), 15 g of Cu/Fe-LDH were placed into 2 L of 10 g/L of OII solution, and the adsorbed amount of OII on Cu/Fe-LDH reached 1306 mg/g.

### Textural characterization results of the thermal treated products

The XRD patterns showed that the heated products of Cu/Fe-LDH were mainly composed by CuO (Fig. s3), while the Fe^3+^ and Cu^2+^ in the spent LDH were reduced by the adsorbed organic contaminants (Fig. 2). S-Cu/Fe-600 was mainly composed of Magnetite and zero-valent Copper, while S-Cu/Fe-800 and S-Cu/Fe-1000 were mainly composed by zero-valent Iron and Copper. The different reduction degrees should result from the different heating temperatures.

**Figure 3:**
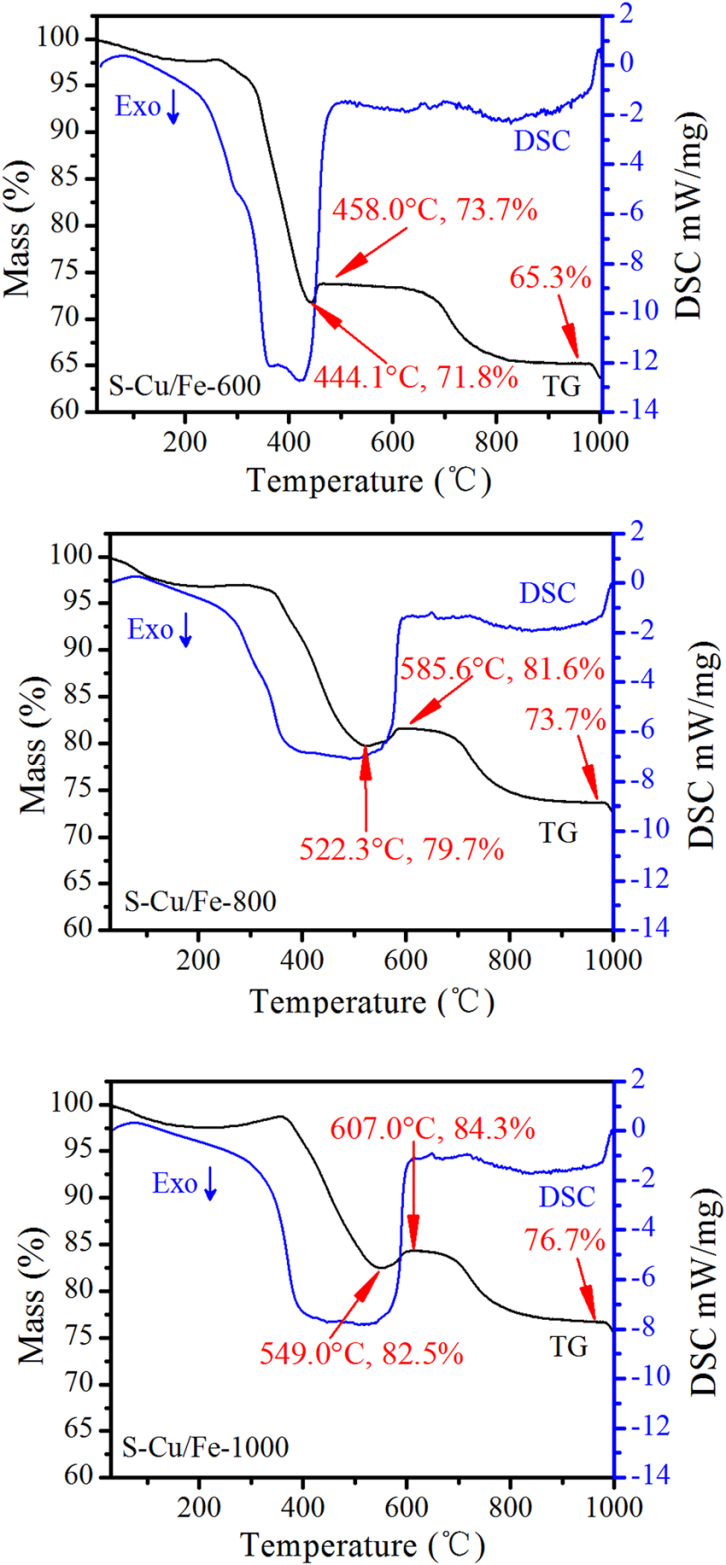
TG analysis of the heated products of the spent Cu/Fe-LDH under air atmosphere.

TG analysis results also confirmed the reduction of the high valence metals. As can be seen in Fig. 3, when heating the samples in an air atmosphere, all samples exhibited a weight gain process at the temperature range from 400 to 700 °C which resulted from the oxidation of the low electronic state of metal species (e.g., Fe^0^ and Cu^0^). TG-DSC curves of each sample presented differences as well. For S-Cu/Fe-600, the temperature of the turning point (the temperature when the rate of weight loss equals that of weight gain) was 444.1 °C, which was smaller than that of S-Cu/Fe-800 (522.3 °C) and S-Cu/Fe-1000 (549.0 °C). In addition, compared to S-Cu/Fe-800 and S-Cu/Fe-1000, S-Cu/Fe-600 had a larger weight loss at temperatures higher than 300 °C and a much sharper DSC exothermic peak. These differences indicate that S-Cu/Fe-600 possesses less of the reduced Fe and/or Cu, which is ascribed to the low reduction degree at the lower heating temperature. For S-Cu/Fe-800 and S-Cu/Fe-1000, the higher turning point and broader DSC exothermic peaks illustrate a higher amount of the reduced metals and/or more kinds of reduced metals.

## Cr(VI) Removal Results

### Effect of initial pH

**Figure 4:**
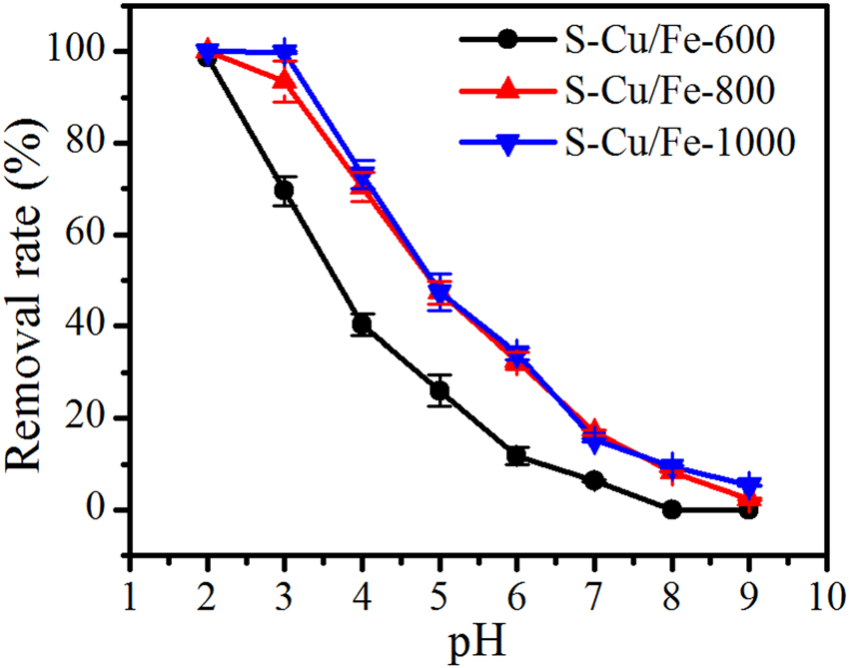
Removal efficiency of Cr(VI) with the variation of pH, the initial concentration of Cr(VI) is 25 mg/L.

Solution pH greatly affected the removal efficiencies of Cr(VI) on S-Cu/Fe-600, S-Cu/Fe-800, and S-Cu/Fe-1000 (Fig. 4). At low solution pH (e.g., pH 2 and 3) Cr(VI) could be almost completely removed, while increasing the pH caused a pronounced decrease of Cr(VI) removal efficiency. S-Cu/Fe-800 and S-Cu/Fe-1000 showed close Cr(VI) removal efficiencies which were much higher than that of S-Cu/Fe-600 at every adopted pH; these results should result from the different reduction degree of Fe^3+^ and Cu^2+^. In addition, the Cr(VI) removal efficiencies showed no dependency on the specific surface areas, as S-Cu/Fe-800 had the largest specific surface area (50.8 m^2^/g) while S-Cu/Fe-1000 presented as the smallest one (18.8 m^2^/g) (Table 1). Thus, the removal of Cr(VI) might be mainly attributed to the reduction of Cr(VI).

**Table 1:** Porosity characterization results of the heated products and carbon materials.

Sample	*S*_BET_	*V*_total_	Sample	*S*_BET_	*V*_total_
	(m^2^/g)	(cm^3^/g)		(m^2^/g)	(cm^3^/g)
S-Fe/Cu-600	40.2	—	Carbon-600	135.2	0.22
S-Fe/Cu-800	50.8	—	Carbon-800	141.3	0.23
S-Fe/Cu-1000	18.8	—	Carbon-1000	133.3	0.23

### Removal kinetics

**Figure 5:**
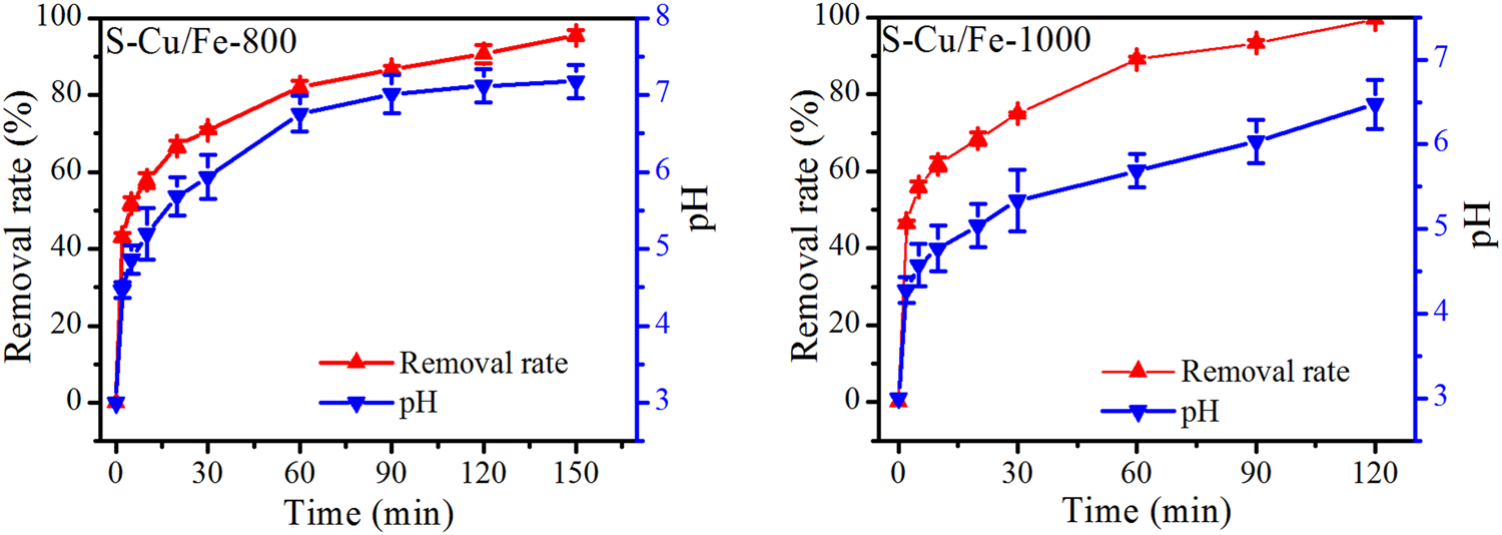
Removal efficiency of Cr(VI) with the variation of time under pH 3. The initial concentration of Cr(VI) is 25 mg/L.

**Figure 6:**
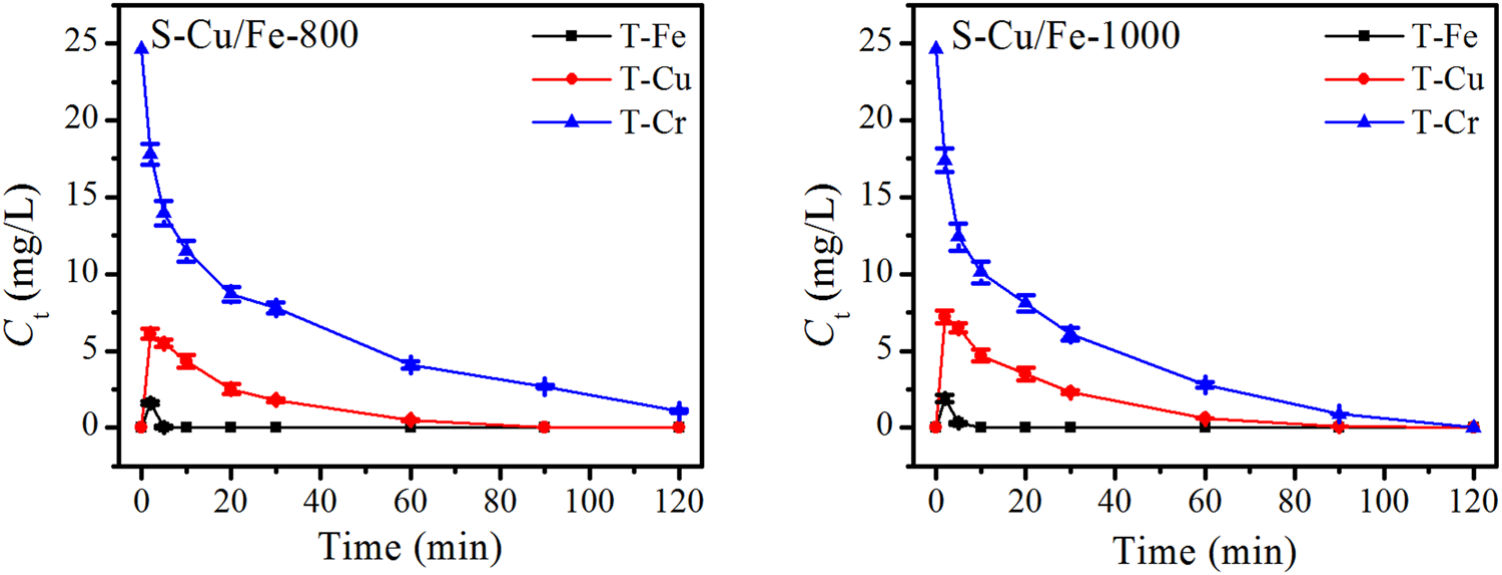
Variation of the concentration of total Fe, Cu, and Cr in the solution under the removal process of Cr(VI) (initial concentration of 25 mg/L) at pH 3.

Due to the fact that S-Cu/Fe-800 and S-Cu/Fe-1000 possessed better Cr(VI) removal efficiencies than S-Cu/Fe-600, these two samples were employed in the subsequent experiments at initial pH of 3. The variation of Cr(VI) removal efficiencies, pH, and the dissolved total metal ions were plotted. Under the adopted experimental conditions, Cr(VI) could be nearly completely removed within 2.5 h, and S-Cu/Fe-1000 presented a higher removal efficiency than S-Cu/Fe-800 (Fig. 5). The removal of Cr(VI) with the variation of time were similar to a Langmuir adsorption model, illustrating that the removal rate of Cr(VI) could be slowed down by some reason as time increased. As reaction duration increased, solution pH increased, and the final pH was higher than 6.5. The increase of pH indicated that a redox reaction occurred within the solutions, and the increase in pH level slows down or inhibits the reduction of Cr(VI)^13^. Therefore, the increase of pH might be one of the reasons causing the decrease of removal rate of Cr(VI) as time increased. Due to the increase of pH, the soluble metal ions (i.e., Cu^2+^, Fe^3+^, and Cr^3+^) would be removed from solution by forming Cu(OH)_2_ (*Ksp* of 4.8 × 10^−20^ ^14^), Fe(OH)_3_ (*Ksp* of 2.79 × 10^−39^ ^15^), and Cr(OH)_3_ (*Ksp* of 7 × 10^−31^ ^16^), which was confirmed by the detection results of the concentrations of total metal ions in solution (Fig. 6). During the Cr(VI) removal process, concentrations of total Fe and Cu increased at first and then decreased to zero, demonstrating that the ions in the solution were Fe^3+^ and Cu^2+^. These two kinds of ions should theoretically be derived from the reaction with Cr(VI) and/or the process of the ions dissolving from the solid itself. As for the concentration of total Cr, it also decreased to zero (or near zero) as time increased, which should result from the adsorption and/or reduction by low valence metals forming Cr(OH)_3_. Notably, one phenomenon should be figured out that though S-Cu/Fe-1000 had better Cr(VI) removal efficiency than that of S-Cu/Fe-800, the increase in pH of its related solution was less than that of the S-Cu/Fe-800 related solution. This result is presumably due to the factor that more Coppers covered the surface of S-Cu/Fe-1000 indicated by the fact that the concentration of total Cu in the S-Cu/Fe-1000 related solution was higher than that in the S-Cu/Fe-800 related solution (Fig. 6).

### Sequential removal experiments

**Figure 7:**
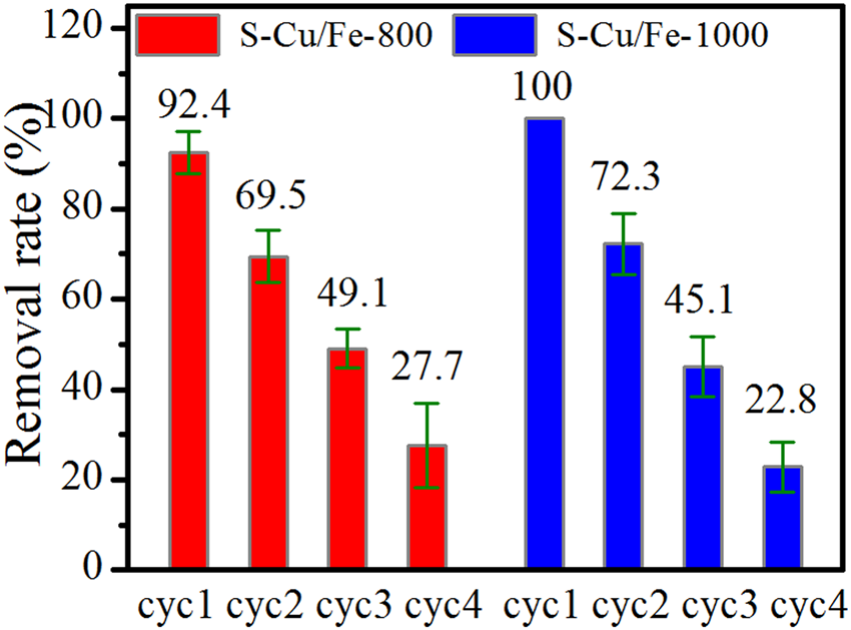
Removal efficiency of Cr(VI) (initial concentration of 25 mg/L) on S-Cu/Fe-800 and S-Cu/Fe-1000 under different cycles at initial pH 3.

To test the maximum removal capacities (for the removal of Cr(VI)) of S-Cu/Fe-800 and S-Cu/Fe-1000 under an initial pH of 3, sequential removal experiments were carried out. After first contacting for 2 h, 25 mg/L of Cr(VI) could almost be completely removed (Fig. 7). After centrifugation, the remnant solid was dispersed in another fresh Cr(VI) solution (pH 3, 25 mg/L) for another 2 h without any treatment. With the increase of cycles, the Cr(VI) removal efficiency of each sample decreased distinctly. According to the cycle experiments, the maximum removal of Cr(VI) on 1 g of S-Cu/Fe-800 and S-Cu/Fe-1000 were respectively higher than 59.7 and 60.1 mg/g, which are higher than some other Cr(VI) removers reported previously such as lignocellulosic wastes^17^. On the other hand, isotherm removal curves of Cr(VI) on S-Cu/Fe-800 and S-Cu/Fe-1000 showed that these materials presented maximal removal capacities of 36–44 mg/g at pH 3 (Fig. s4) under the tested concentration range of 20 to 150 mg/L, which are comparable to that of biochar-supported Fe^0^ (36–56 mg/g) at pH 3^18^ and bimetallic iron-silver zero-valent nanoparticles (42–56 mg/g) determined at pH 2^19^ (generally a lower pH generated a higher Cr(VI) reduction capacity).

### Removal mechanism

The above results indicated the presence of a redox reaction in the Cr(VI) removal process, but could not clarify the removal mechanism. Based on the redox potential (herein mark as *E* ^0^ or *E*) of Cr(VI)/Cr^3+^ (equation 1, at pH 3)^20^, Fe^3+^/Fe^0^ (equation 2)^21^, Fe^3+^/Fe^2+^ (equation 3)^22^, Fe^2+^/Fe^0^ (equation 4)^23^, Cu^2+^/Cu^+^ (equation 5), Cu^2+^/Cu^0^ (equation 6), Cu^+^/Cu^0^ (equation 7)^24^, and H^+^/H_2_ (equation 8), the reactions between Cr(VI) and Fe^0^/Fe^2+^/Cu^0^/Cu^+^ (equations 9/10/11/12), Fe^0^ and Cu^2+^/Cu^+^/H^+^ (equations 13/14/15) can theoretically spontaneously happen when these low valence metals/metal ions (i.e., Fe^0^/Fe^2+^/Cu^0^/Cu^+^) exist (*E* > 0).

1 $${{\rm{Cr}}}_{2}{{{\rm{O}}}_{7}}^{2-}+14{{\rm{H}}}^{+}+6{{\rm{e}}}^{-}\leftrightarrow 2{{\rm{Cr}}}^{3+}+7{{\rm{H}}}_{2}{\rm{O}}\quad \quad \quad \quad \quad {E}^{0}=+1.33\,V\,$$

2 $${{\rm{Fe}}}^{3+}+3{{\rm{e}}}^{-}\leftrightarrow {{\rm{Fe}}}^{0}\quad \quad \quad \quad \quad \quad {E}^{0}=-0.04\,V$$

3 $${{\rm{Fe}}}^{3+}+{{\rm{e}}}^{-}\leftrightarrow {{\rm{Fe}}}^{2+}\quad \quad \quad \quad \quad \quad \quad {E}^{0}=+0.77\,V$$

4 $${{\rm{Fe}}}^{2+}+2{{\rm{e}}}^{-}\leftrightarrow {{\rm{Fe}}}^{0}\quad \quad \quad \quad \quad \quad {E}^{0}=-0.44V$$

5 $${{\rm{Cu}}}^{2+}+{{\rm{e}}}^{-}\leftrightarrow {{\rm{Cu}}}^{+}\quad \quad \quad \quad \quad \quad {E}^{0}=+0.16\,V$$

6 $${{\rm{Cu}}}^{2+}+2{{\rm{e}}}^{-}\leftrightarrow {{\rm{Cu}}}^{0}\quad \quad \quad \quad \quad \quad {E}^{0}=+0.34\,V$$

7 $${{\rm{Cu}}}^{+}+{{\rm{e}}}^{-}\leftrightarrow {{\rm{Cu}}}^{0}\quad \quad \quad \quad \quad \quad {E}^{0}=+0.52\,V$$

8 $$2{{\rm{H}}}^{+}+2{{\rm{e}}}^{-}\leftrightarrow {{\rm{H}}}_{2}\quad \quad \quad \quad \quad \quad {E}^{0}=0.00\,V$$

9 $${{\rm{Cr}}}_{2}{{{\rm{O}}}_{7}}^{2-}+14{{\rm{H}}}^{+}+3(2){{\rm{Fe}}}^{0}\leftrightarrow 2{{\rm{Cr}}}^{3+}+7{{\rm{H}}}_{2}{\rm{O}}+3{{\rm{Fe}}}^{2+}(2{{\rm{Fe}}}^{3+})\,E=+1.77(+1.37)V$$

10 $${{\rm{Cr}}}_{2}{{{\rm{O}}}_{7}}^{2-}+14{{\rm{H}}}^{+}+6{{\rm{Fe}}}^{2+}\leftrightarrow 2{{\rm{Cr}}}^{3+}+7{{\rm{H}}}_{2}{\rm{O}}+6{{\rm{Fe}}}^{3+})\quad \quad \quad \quad \quad \quad E=+0.56V$$

11 $${{\rm{Cr}}}_{2}{{{\rm{O}}}_{7}}^{2-}+14{{\rm{H}}}^{+}+3(6){{\rm{Cu}}}^{0}\leftrightarrow 2{{\rm{Cr}}}^{3+}+7{{\rm{H}}}_{2}{\rm{O}}+3{{\rm{Cu}}}^{2+}(6{{\rm{Cu}}}^{+})\,E=+0.99(+0.81)V$$

12 $${{\rm{Cr}}}_{2}{{{\rm{O}}}_{7}}^{2-}+14{{\rm{H}}}^{+}+6{{\rm{Cu}}}^{+}\leftrightarrow 2{{\rm{Cr}}}^{3+}+7{{\rm{H}}}_{2}{\rm{O}}+6{{\rm{Cu}}}^{2+}\quad \quad \quad \quad \quad \quad E=+1.17\,V$$

13 $${{\rm{Fe}}}^{0}\,+2(1){{\rm{Cu}}}^{2+}\leftrightarrow {{\rm{Fe}}}^{2+}+2{{\rm{Cu}}}^{+}({{\rm{Cu}}}^{0})\quad \quad \quad \quad \quad \quad E=+0.60(+0.78)V$$

14 $${{\rm{Fe}}}^{0}+2{{\rm{Cu}}}^{+}\leftrightarrow {{\rm{Fe}}}^{2+}+2{{\rm{Cu}}}^{0}\quad \quad \quad \quad \quad \quad E=+0.96\,V$$

15 $${{\rm{Fe}}}^{0}+2{{\rm{H}}}^{+}\leftrightarrow {{\rm{Fe}}}^{2+}+{{\rm{H}}}_{2}\quad \quad \quad \quad \quad \quad E=+0.44\,V$$

According to equations (9) and (11), when Fe^0^ and Cu^0^ reacted with Cr(VI), they preferred to form Fe^3+^ and Cu^2+^ rather than Fe^2+^ and Cu^+^. In addition, Fe^2+^ and Cu^+^ can further react with Cr(VI) (equations 10 and 12). This is the reason that all the Fe and Cu were precipitated after Cr(VI) removal (under a neutral pH).

**Figure 8:**
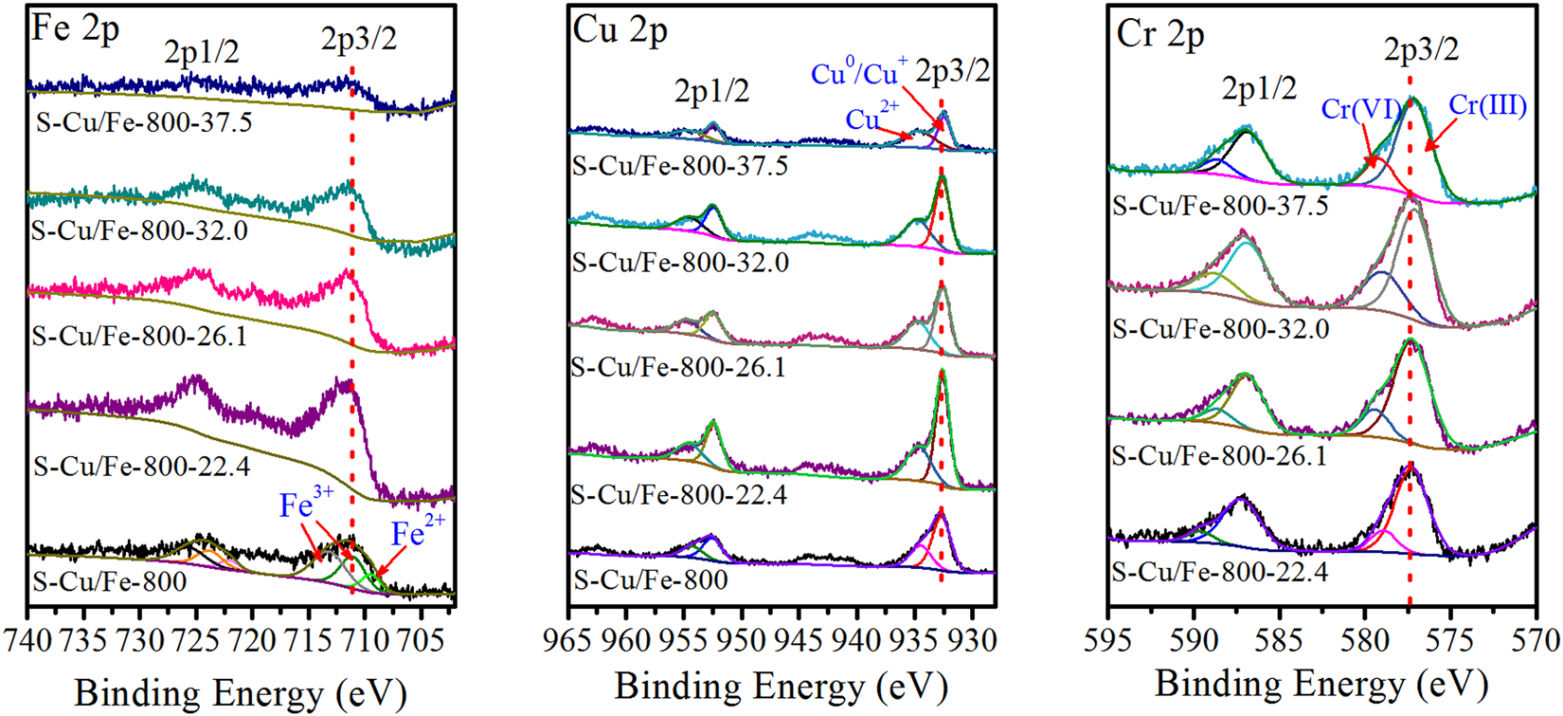
Fe 2p, Cu 2p, and Cr 2p XPS spectra of the S-Cu/Fe-800 and the used S-Cu/Fe-800; the used S-Cu/Fe-800 was obtained from the treatment of Cr(VI) under different Cr(VI) concentrations (25–100 mg/L), and hereby named as S-Cu/Fe-800-X herein (the ‘X’ represents the removed amount of Cr(VI)).

To explore the reactions between the low valence metals/metal ions and Cr(VI), and to clarify the contribution of adsorption and reduction, XPS characterization of the used S-Cu/Fe-800 generated from the treatment of Cr(VI) (with various Cr(VI) concentrations from 25–100 mg/L) was carried out. Fe 2p spectra showed that in S-Cu/Fe-800, the iron species were mainly Fe^2+^ and Fe^3+^, while Fe^0^ was not detected (Fig. 8). Xi *et al*. considered the reason for this is that the probing depth of the XPS is limited (only 2–5 nm)^25^. With an increase in the removed amount of Cr(VI) by S-Cu/Fe-800, the binding energy of the main peak of Fe 2p3/2 increased, which illustrated that Fe^2+^ was oxidized. Therefore, the reactions between Fe^0^/Fe^2+^ and Cr(VI) (equations 9 and 10) should happen.

From Cu 2p spectra, peaks belonging to Cu^0^/Cu^+^ and Cu^2+^ could be observed^26^. After treatment with Cr(VI), the Cu^2+^ peak became more obvious, demonstrating that a reaction between Cu^0^/Cu^+^ and Cr(VI) also took place (equations 11 and 12). On the other hand, the binding energy of the main peak of Cu 2p3/2 decreased with an increase in the removal amount of Cr(VI) by S-Cu/Fe-800, indicating that some Cu^+^ and/or Cu^0^ could be generated. The increase of Cu^0^/Cu^+^ might be due to the reaction with Cr(VI) (equation 11) and/or Fe^0^ (equations 13 and 14). The generation of Cu^+^ indicates that the used S-Cu/Fe-800 can still be used as the reductant for Cr(VI).

During the reduction of Cr(VI), consumption of H^+^ happened (equations 9–12, and 15), which then caused the increase of solution pH. The increase of pH (i.e., the decrease of the concentration of H^+^) further resulted in the slowing down of the reduction rate of Cr(VI). On the other hand, according to Yoon *et al*. the redox potential of Cr(VI)/Cr^3+^ decreased with pH increase, with a shifting of 138 mV per pH unit^20^. Therefore, these two aspects forced the reduction curves of Cr(VI) to a Langmuir type with time increase (Fig. 5). Notably, the consumption of H^+^ should be mainly caused by the reduction of Cr(VI), as equation (15) is not a preferential reaction when Cr(VI) and Cu^2+^/Cu^+^ exist (according to the redox potentials).

The Cr 2p spectra showed that both Cr(VI) and Cr(III) presented on the surface of the used S-Cu/Fe-800, which demonstrated that the removal of Cr(VI) was the combination of adsorption with reduction, and the latter played the dominant role. According to the peak areas, about 71.4–83.3% of the removed Cr(VI) is contributed to reduction. Increasing the initial concentration of Cr(VI) from 25 to 100 mg/L (the removal amount of Cr(VI) increased from 22.5 to 37.5 mg/g) caused the contribution ratio of adsorption to increase from 16.7 to 28.6%. The decrease of the contribution ratio of reduction might be ascribed to the increase of solution pH. On the other hand, the binding energy of the main peaks of Cr 2p decreased slightly as the removal amount of Cr(VI) increased. This result indicated that with an increase in the adsorbed amount of Cr, the interaction between the S-Cu/Fe-800 surface and Cr became weaker.

### Disposing the wastes

**Figure 9:**
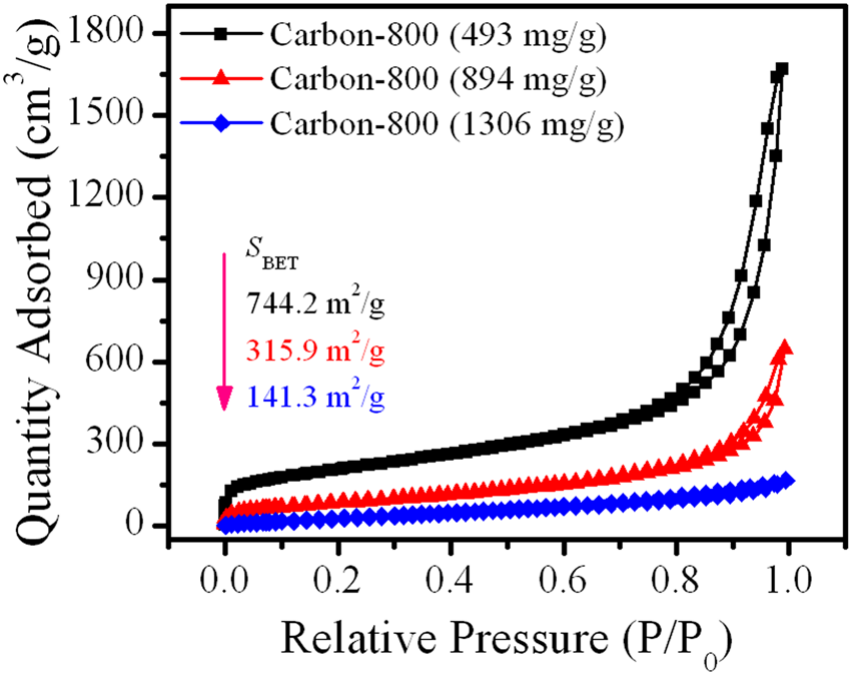
N_2_ adsorption-desorption property of the carbon materials obtained from the spent Cu/Fe-LDH with various adsorbed amounts of OII via carbonizing at 800 °C and acid washing. 493, 894, and 1306 mg/g represent the adsorbed amount of OII on the Cu/Fe-LDH.

The samples after the removal of Cr(VI) (i.e., the spent S-Cu/Fe-600/800/1000) can also be utilized to obtain porous carbon materials by acid washing. N_2_ adsorption-desorption was employed to investigate the surface structures of the porous carbon materials generated from used/spent S-Cu/Fe-600–1000 via acid washing. Results showed that S-Cu/Fe-600, S-Cu/Fe-800, and S-Cu/Fe-1000 had specific surface areas of 40.2, 50.8, and 18.8 m^2^/g, respectively; by acid washing, surface area could respectively increase to 135.2, 141.3, and 133.3 m^2^/g, respectively (Table 1). However, even though the surface areas increased greatly after acid washing, the surface areas were still relatively small which poses problems. The low surface area might be attributed to the excessive amount of OII adsorbed on Cu/Fe-LDH (1306 mg/g), which decreases the dispersity of OII on the surface of the LDH. To examine the presupposition, spent Cu/Fe-LDH with various adsorbed amounts of OII (493, 894, and 1306 mg/g) were prepared, and were subsequently used to prepare carbon materials under a temperature of 800 °C. The results showed that the increase of the adsorbed OII caused a pronounced decrease of specific surface area of the resulting carbon materials (from 744.2 to 141.3 m^2^/g) (Fig. 9). A low adsorbed amount of OII would produce a large amount of mesopores (judged by hysteresis loop) and macropores (judged by the pronounced increase of the adsorbed amount of N_2_ at high relative pressure) as well. SEM characterization results of these carbon materials showed that they still retained some fine layered morphology (Fig. s1). The SEM pictures also revealed that there were still some components of the heated products of Cu/Fe-LDH retaining in the final products (i.e., the carbon materials), which may be one of the reasons that caused the small specific areas of Carbon-800 (894) and Carbon-800 (1306). Therefore, the spent Cu/Fe-LDH can be utilized to prepare porous carbon materials as well, but one must control the adsorbed amount of organic contaminants and wash out all the components of the heated product of Cu/Fe-LDH. Notably, the heated products (800 °C) generated from spent Cu/Fe-LDH with adsorbed OII amounts of 493 and 894 mg/g presented good Cr(VI) removal properties as well (Fig. s5).

On the other hand, the waste metals solution generated from acid washing can still be used to synthesize LDH with well crystallinity (Fig. s6), which indicated that all the components of the spent Cu/Fe-LDH can be recycled and the above waste recycling method is sustainable. In the whole recycling process, about all the Cu and Fe elements in the original Cu/Fe-LDH can be recycled to regenerate new Cu/Fe-LDH. As indicated by Fig. 6, after Cr(VI) treatment there are not any Fe and Cu ions left in solution, which means that both Fe and Cu ions finally transfer to solids. Additionally, when the Cr(VI) reductant loses its reducing properties which means the final pH of the treated Cr(VI) solution will be below 6.5 (*Ksp*_Cu(OH)2_ = 4.8 × 10^−20^ and *Ksp*_Fe(OH)3_ = 2.79 × 10^−39^) and some Cu^2+^ will be leached in solution, one still can modulate the solution pH to 6.5 or higher value to precipitate Cu^2+^ and Fe^3+^ ions. Therefore, all the Cu and Fe will be leached to form double metal solution for regenerating new Cu/Fe-LDH when using acid to dissolve the used S-Cu/Fe-600/800/1000. In the Cu/Fe-LDH regenerating process, about 85% of Cu element can be recycled to the regenerated Cu/Fe-LDH, and all Fe^3+^ can form solid of Cu/Fe-LDH and/or Fe(OH)_3_. Notably, adsorption isotherm results show that the regenerated Cu/Fe-LDH still presents high adsorption performance toward organic contaminant OII (Fig. s7), which further bolsters the cycled utilization of spent LDHs.

## Conclusions

The spent Cu/Fe-LDH rich in organic contaminants can be recycled by converting to Cr(VI) reductant and porous carbon material, and the generated waste metal solution is reusable for LDH synthesis. The obtained reductant exhibited good reductive activity toward Cr(VI). Both adsorption and reduction contributed in the process of the removal of Cr(VI), and reduction played the dominant role. The reduction of Cr(VI) was caused by the reactions between Cr(VI) and Fe^0^/Fe^2+^/Cu^0^/Cu^+^. These reactions also caused the increase of solution pH, which greatly favored the precipitation of the metal ions. On the other hand, the specific surface area of the obtained porous carbon material is tunable by controlling the adsorbed amount of organic contaminants. This study provides a cyclic utilization of all the elements of LDHs type organic-inorganic solid wastes. The results from this study are expected to be applicable to further studies on the recycling of other similar organic-inorganic solid wastes. This study teaches us that one should carefully consider which material to be used in an application to reduce waste and allow for simple recycling. One can make the waste generated subsequently easy to recycle via designing proper initial products.

## Experimental Details

### Materials

Fe(NO_3_)_3_·9H_2_O (98%), Cu(NO_3_)_2_·3H_2_O (98%), K_2_Cr_2_O_7_ (98%), NaOH, and HCl were purchased from Guangzhou chemical reagent Co. Ltd., and used as received. Orange II (C_16_H_11_N_2_NaO_4_S, > 85%) was purchased from Aladdin-E.

### Preparation of Cu/Fe-LDH

Cu/Fe-LDH with NO_3_^−^ located in the interlayers of its structure was synthesized using a conventional co-precipitation method^27–29^. First, two 500 mL solutions were prepared, one containing 0.25 mol of Fe(NO_3_)_3_·9H_2_O (98%) and 0.5 mol of Cu(NO_3_)_2_·3H_2_O (98%), the other containing 0.5 mol of sodium hydroxide. Then, these two solutions were added dropwise into a beaker (initially containing 200 mL of ultrapure water) under nitrogen protection and vigorous stirring at room temperature. The pH was maintained at 5.5 ± 0.1 using Chroma CPH-2 automatic pH controller. The generated heavy gel was crystallized at 75 °C for 12 h under nitrogen atmosphere. After that, the product was centrifuged and washed with ultrapure water until the supernatant pH was near 7 before drying at 80 °C for 24 h.

### Adsorption of OII on Cu/Fe-LDH

OII (C_16_H_11_N_2_NaO_4_S, purchased from Aladdin-E, > 85%) adsorption isotherm experiments were carried out by a batch adsorption method in a shaker (200 rpm) under room temperature (25 °C). About 50 mg of Cu/Fe-LDH was dispersed in 50 mL of OII solutions with varying concentrations (50–1000 mg/L). After the given contact time for adsorption (12 h), the supernatant was collected by centrifugation and then analyzed at 485 nm using a UV–Vis spectrophotometer (Perking Elmer Precisely Lambda 850) to determine the remnant concentration of OII. To generate spent Cu/Fe-LDH with rich adsorbed amounts of OII, 15 g of Cu/Fe-LDH were added into 2 L of 10 g/L OII solution, and the generated spent Cu/Fe-LDH were collected as raw material for the subsequent experiments after drying at 80 °C.

### Thermal treatment of the spent Cu/Fe-LDH

The spent Cu/Fe-LDH were loaded into a crucible, and heated in a temperature-controlled tubular furnace to the desired temperature (600–1000 °C) under the protection of nitrogen. The heating rate was 5 °C/min, and the samples were maintained at the target temperature for 3 h. After cooling the samples to room temperature under nitrogen atmosphere, the obtained materials were milled. These products with particle sizes between 100 and 200 meshes were collected and stored in a desiccator over silica gel in a nitrogen atmosphere. The materials obtained under the heating temperatures of 600, 800, and 1000 °C herein were referred to as S-Cu/Fe-600, S-Cu/Fe-800, and S-Cu/Fe-1000, respectively. For comparison, Cu/Fe-LDH was also heated under the same conditions, and the resulting material was named as Cu/Fe-x (‘x’ represents the heating temperature).

### Cr(VI) removal experiments

Cr(VI) (K_2_Cr_2_O7, 98%) removal experiments were conducted using glass vials in a batch equilibrium technique in aqueous solution at pH 2–9 and room temperature. Excepting some special cases, the remover dosage was 1 g/L, Cr(VI) concentration was 25 mg/L, pH was 3, and the contact time was 12 h. At the given contact time, the reaction solution was sampled and filtered through 0.22 μm membrane. Concentrations of Cr (VI) were quantified at 540 nm, using 1, 5-diphenylcarbazide in acid solution as the complexing agent^30, 31^. On the other hand, total concentrations of dissolved metals (Cr, Fe, and Cu) were determined using atomic absorbance spectrometer (AAS, PE AAnalyst 400).

To investigate the maximum removal efficiency of Cr(VI) by the obtained heated products (S-Cu/Fe-800 and S-Cu/Fe-1000 were taken as examples), sequential removal experiments were carried out. 0.1 g of S-Cu/Fe-800 or S-Cu/Fe-1000 was dispersed in 100 mL of 25 mg/L Cr(VI) solution with an initial pH of 3. After contacting for 2 h, the remnant solid was collected by centrifugation. Then, the collected solid was dispersed in 100 mL of 25 mg/L Cr(VI) solution (pH 3) again without any treatment. The experiments were repeated several times.

### Disposing the spent S-Cu/Fe-600/800/1000

The spent S-Cu/Fe-600, S-Cu/Fe-800, and S-Cu/Fe-1000 generated from the removal of Cr(VI) were used to prepare porous carbon materials via acid washing. These samples were washed with HCl (2 M) for 6 h under magnetic stirring to remove the inorganic metal/metal oxide phase. After that, the generated carbon materials were further washed several times with ultrapure water and then dried at 80 °C overnight. The carbon materials obtained from S-Cu/Fe-600, S-Cu/Fe-800, and S-Cu/Fe-1000 are herein referred to as Carbon-600, Carbon-800, and Carbon-1000, respectively.

On the other hand, the generated waste metals solution was reused for synthesizing LDH.

### Characterization methods

XRD patterns of the heated products were measured on a Bruker D8 ADVANCE X-ray diffractometer using Cu K*α* radiation operating at 40 kV and 40 mA. The patterns were recorded over the 2*θ* range from 3 to 80° with a scan speed of 3°/min using a bracket sample holder.

The morphology of the samples was recorded by a Hitachi SU8010 field-emission scanning electron microscope. The sample powders were firstly stuck to conductive silver adhesives on a glass slide, and then sprayed with a thin layer of conductive carbon to enhance the conductivity before observation.

TG analysis was carried out on a Netzsch STA 490 PC thermal analyzer at a heating rate of 10 °C/min in the temperature range of 30–1000 °C under air atmosphere. The flow rate was 60 mL/min.

XPS spectra were recorded using a K*α* X-ray photoelectron spectrometer (Thermo Fisher Scientific, UK) with a monochromatic Al K*α* X-ray source. All spectra were calibrated using a C 1s peak with a fixed value of 284.8 eV.

Nitrogen adsorption-desorption measurements were determined at 77 K using an ASAP 2020 Surface Area & Pore Size Analyzer (Micromeritics Instrument Corporation). Prior to measurement, the samples were degassed in a vacuum at 120 °C for 12 h. Specific surface area was calculated using the Brunauer-Emmett-Teller (BET) method. Total pore volume was determined from the adsorbed amount of nitrogen at *P*/*P*_0_ = 0.99.

### Data availability statement

All data generated or analysed during this study are included in this published article (and its Supplementary Information files).

## Electronic supplementary material

Supplementary materials
