# Supplementary material for: Converting Spent Cu/Fe Layered Double Hydroxide into Cr(VI) Reductant and Porous Carbon Material
Source: Sci Rep. 2017 Aug 4;7:7277. doi: 10.1038/s41598-017-07775-8 (PMC5544766; doi:10.1038/s41598-017-07775-8)
Supplement: Supplementary file 1 — Supplementary materials [file 41598_2017_7775_MOESM1_ESM.pdf]

Converting Spent Cu/Fe Layered Double Hydroxide into Cr(VI) Reductant and Porous Carbon Material

Minwang Laipan[a,b,c], Haoyang Fu[a,b], Runliang Zhu[a,]*, Luyi Sun[c,]*, Jianxi Zhu[a], Hongping He[a,b]

[a] *CAS Key Laboratory of Mineralogy and Metallogeny / Guangdong Provincial Key Laboratory of Mineral Physics and Materials, Guangzhou Institute of Geochemistry, Chinese Academy of Sciences (CAS), Guangzhou 510640, China*

[b] *University of Chinese Academy of Sciences, Beijing 100049, China*

[c.] *Department of Chemical & Biomolecular Engineering, Polymer Program, Institute of Materials Science, University of Connecticut, Storrs, CT 06269, USA*

* Corresponding author

Dr. Runliang Zhu, E-mail: zhurl@gig.ac.cn, Phone: +86-020-85297603

Dr. Luyi Sun, E-mail: luyi.sun@uconn.edu, Phone: +1-860-486-6895

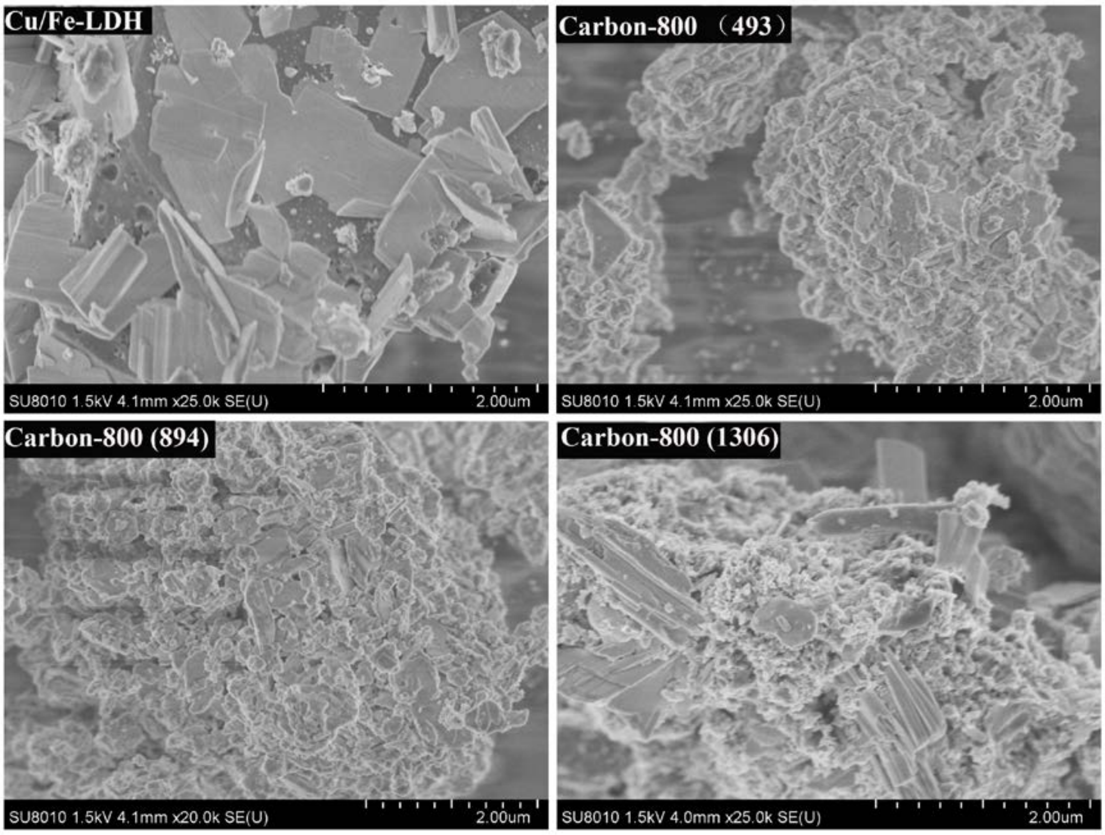

Figure s1. SEM images of Cu/Fe-LDH and carbon materials obtained from the spent Cu/Fe-LDH with various adsorbed amounts of OII via carbonizing at 800°C and acid washing. 493, 894, and 1306 mg/g represent the adsorbed amount of OII on the Cu/Fe-LDH

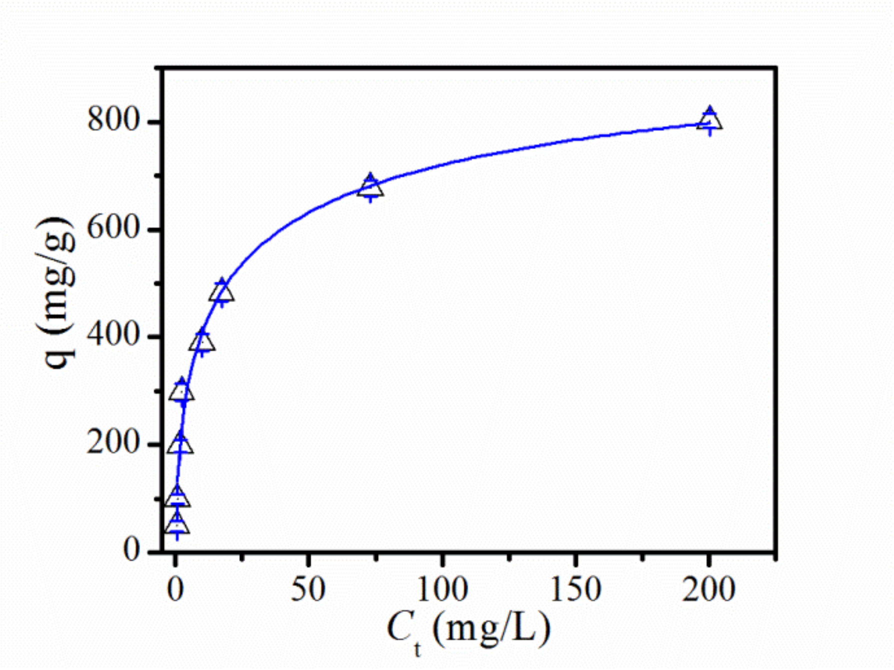

Figure s2. Adsorption isotherm of Orange II on Cu/Fe-LDH under 25°C

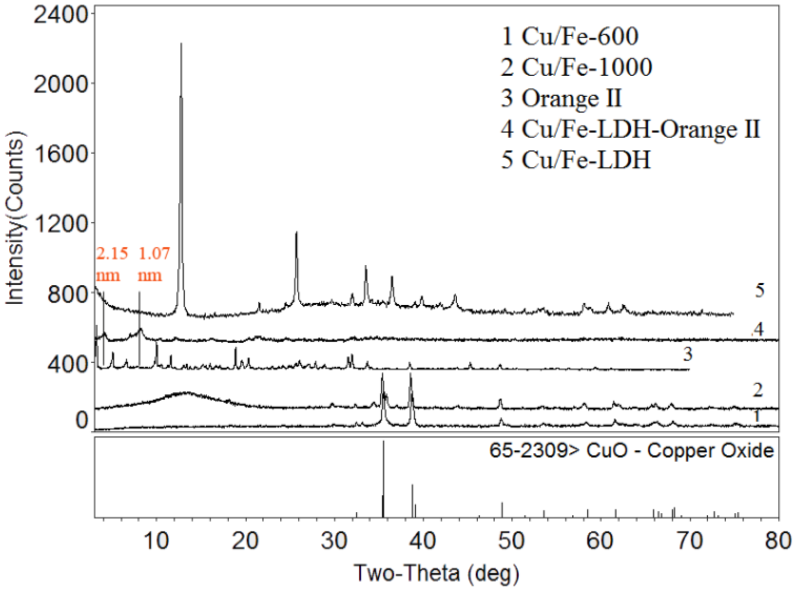

Figure s3. XRD patterns of Orange II, spent Cu/Fe-LDH (Cu/Fe-LDH-Orange II), Cu/Fe-LDH, Fe/Cu-600, and Fe/Cu-1000.

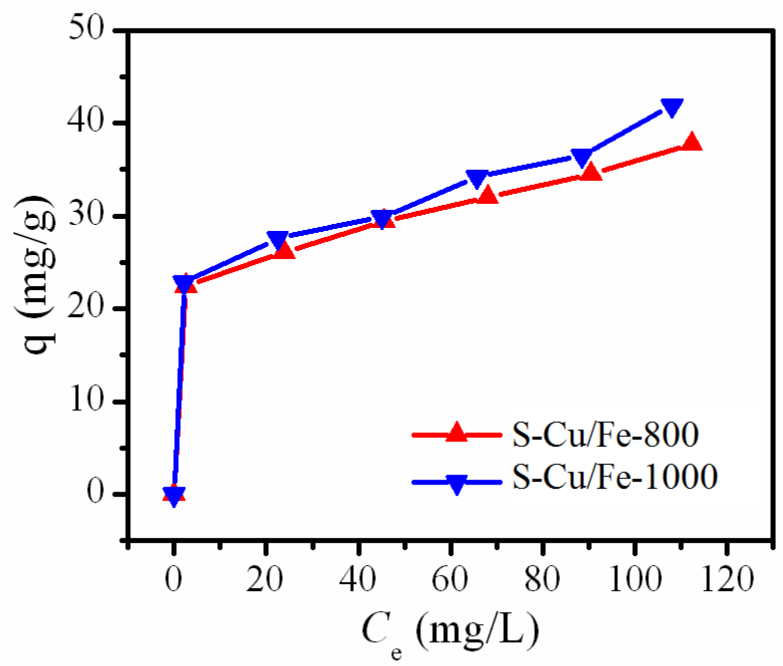

Figure s4. Isotherm removal curves of Cr(VI) on S-Cu/Fe-800 and S-Cu/Fe-1000 at pH 3 and room temperature (Initial concentrations of Cr(VI) vary from 20 to 150 mg/L)

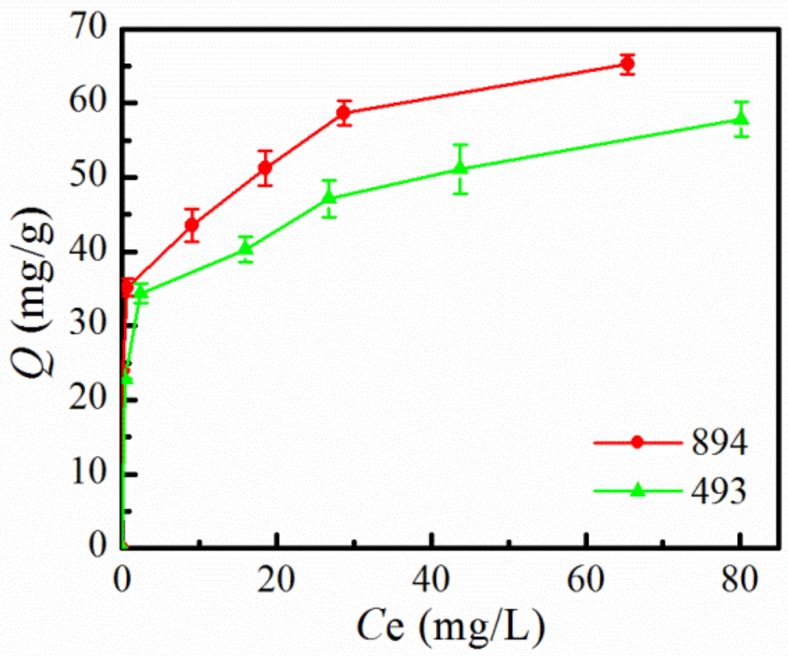

Figure s5. Cr(VI) removal isotherms (25°C) on S-Cu/Fe-800 generated from the spent Cu/Fe-LDH loaded with different OII amount (894 and 493 mg/g)

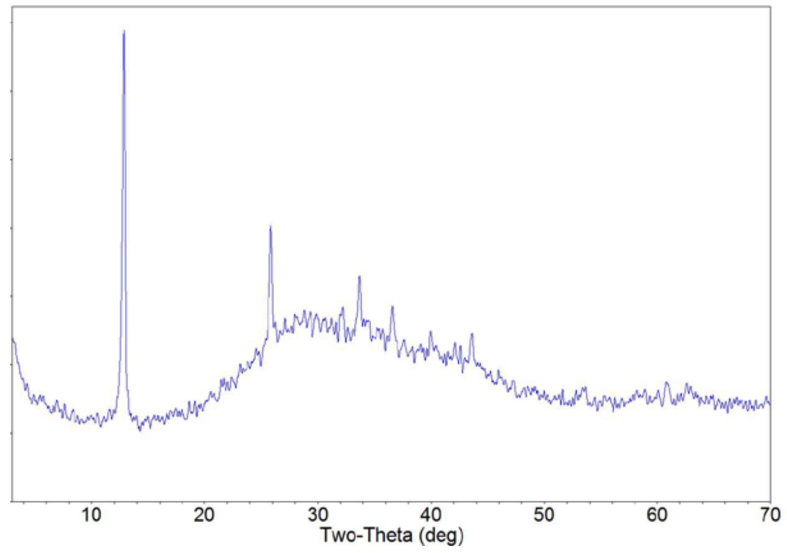

Figure s6. XRD patterns of LDH prepared from waste metal solution (Synthesis at pH 6.0, aging at 75°C for 24 h)

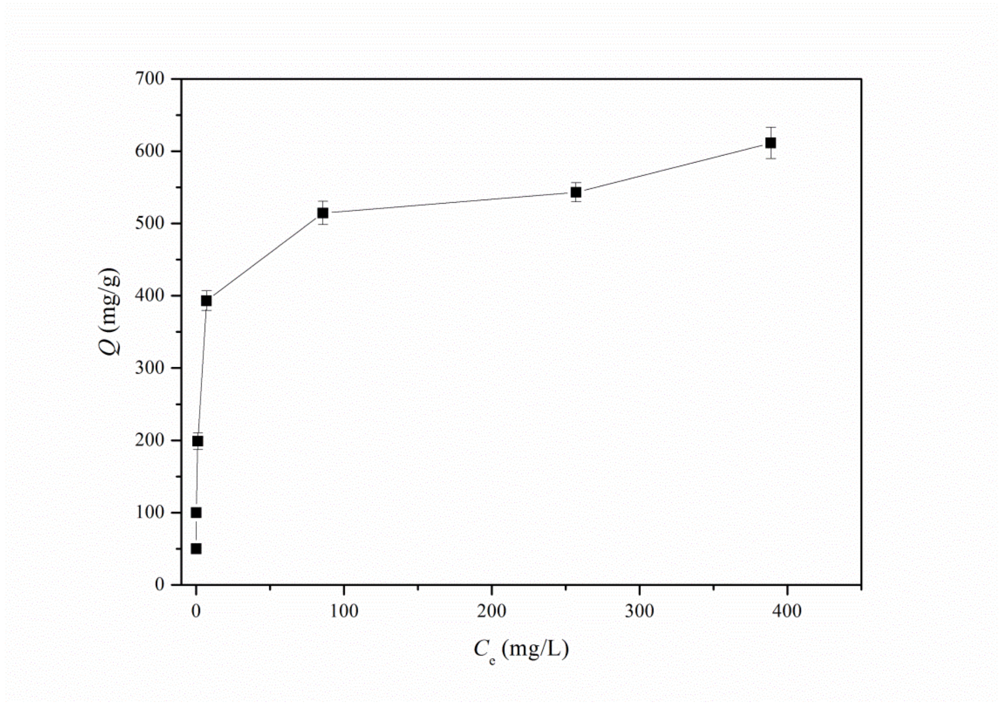

Figure s7. Adsorption isotherm of Orange II on regenerated Cu/Fe-LDH under 25°C
